# Bottom-Up Synthesis and Sensor Applications of Biomimetic Nanostructures

**DOI:** 10.3390/ma9010053

**Published:** 2016-01-18

**Authors:** Li Wang, Yujing Sun, Zhuang Li, Aiguo Wu, Gang Wei

**Affiliations:** 1 College of Chemistry, Jilin Normal University, Haifeng Street 1301, Siping 136000, China; 2 State Key Laboratory of Electroanalytical Chemistry, Changchun Institute of Applied Chemistry, Chinese Academy of Sciences, Renmin Street 5625, Changchun 130022, China; yjsun@ciac.jl.cn (Y.S.); zli@ciac.jl.cn (Z.L.); 3 Key Laboratory of Magnetic Materials and Devices & Division of Functional Materials and Nanodevices, Ningbo Institute of Material Technology and Engineering, Chinese Academy Sciences, Ningbo 315201, China; aiguo@nimte.ac.cn; 4 Faculty of Production Engineering, University of Bremen, Am Fallturm 1, D-28359 Bremen, Germany

**Keywords:** biomolecules, biomimetic, self-assembly, nanoparticles, nanostructures, sensor

## Abstract

The combination of nanotechnology, biology, and bioengineering greatly improved the developments of nanomaterials with unique functions and properties. Biomolecules as the nanoscale building blocks play very important roles for the final formation of functional nanostructures. Many kinds of novel nanostructures have been created by using the bioinspired self-assembly and subsequent binding with various nanoparticles. In this review, we summarized the studies on the fabrications and sensor applications of biomimetic nanostructures. The strategies for creating different bottom-up nanostructures by using biomolecules like DNA, protein, peptide, and virus, as well as microorganisms like bacteria and plant leaf are introduced. In addition, the potential applications of the synthesized biomimetic nanostructures for colorimetry, fluorescence, surface plasmon resonance, surface-enhanced Raman scattering, electrical resistance, electrochemistry, and quartz crystal microbalance sensors are presented. This review will promote the understanding of relationships between biomolecules/microorganisms and functional nanomaterials in one way, and in another way it will guide the design and synthesis of biomimetic nanomaterials with unique properties in the future.

## 1. Introduction

Nanotechnology is the most promising technique to study the structure and property of substance with a size between 1 and 100 nm. The aim of nanotechnology is to create unique and functional nanomaterials and nanodevices with atoms, molecules, and nanoscale building blocks by the controllable fabrication. For the preparation of nanostructures and nanomaterials, two main ways, top-down and bottom-up, have been widely used [1,2,3,4]. Because of the restrictions of physical conditions and technique, it is difficult to produce nanoscale structures and materials with the top-down techniques. For example, the most common top-down techniques based on photolithography have low resolution ascribed to the limitation of optical diffraction effects (0.2–0.5 µm). Other lithographic techniques based on scanning probe microscopy (dip-pen nanolithography) [5,6], microcontact printing [7], and nanoimprint lithography [8], have been developed for creating different nanostructures, but these techniques are generally neither cost-effective nor time-effective. Especially, the resolution below the 100 nm range is not easily achievable in some cases.

To solve these problems, more and more attempts based on bottom-up techniques have been performed to make functional nanomaterials [9,10,11]. The bottom-up approach provides the possibility to create hierarchical and ordered nanostructures and nanomaterials by taking advantages of the physicochemical interactions and self-assembly of molecules and nanoscale building blocks. In these techniques, the molecular especially the biomolecular building blocks play very important roles for the finial creation of nanomaterials with desired functions and properties [12]. Biomolecules like DNA, protein, peptide, enzyme, and others represent nanoscale materials with encoded structural and functional information. For example, biomolecules themselves can mediate the formation of nanoparticles (NPs) or connect NPs to 1D, 2D, and 3D nanomaterials by using their functional groups [13,14]. In addition, biomolecules have the ability to form supramolecular nanostructures by the molecular self-assembly [15,16,17], and the formed supramolecular nanostructures can be the excellent building blocks or templates to further prepare functional nanomaterials [18,19]. Compared to the inorganic building blocks like metallic nanowires, biomolecules and their supra-structures preserve the unique nanoscale effect, molecular linear structure, physicochemical stability, self-assembly ability, and molecular recognition, and have being widely used in the fields of materials science, biophysical science, analytical science, and biomedical engineering [20].

Previously, several research groups have provided their perspectives and views on how to create biomolecule-based nanomaterials and nanostructures [21,22,23,24]. For instance, Sotiropoulou *et al.* presented an overview on the biotemplated nanostructured materials [21], and Willner *et al.* summarized the applications of DNA-NP and DNA-carbon nanotube hybrid systems for sensing [22]. In this review, we summarized the studies on the design, fabrications, and sensor applications of biomolecule and microorganism based nanostructures. The strategies for creating different nanostructures by using DNA, protein, peptide, virus, bacterium, and other biomolecules or microorganisms are introduced. In addition, the potential applications of the synthesized nanostructures in colorimetry, fluorescence, surface plasmon resonance (SPR), surface-enhanced Raman scattering (SERS), electrical resistance, electrochemistry (EC), and quartz crystal microbalance (QCM) sensors are presented. This work will promote the understanding of the relationships between biomolecules or microorganisms and functional nanomaterials in one way, and in another way it will guide the design and synthesis of biomimetic nanomaterials with unique and desired properties and functions.

## 2. Design and Synthesis of Biomimetic Nanostructures

Biomolecules and microorganisms show great potential for the preparation of functional nanomaterials and nanodevices. In one way, the biomolecules or microorganisms themselves can be used as templates and building blocks for the assembly of NPs to form complex nanostructures. In another way, some biomolecules can self-assemble into ordered suprastructures and be further used for the fabrication of functional nanomaterials. In this part, we would like to introduce briefly the examples by using some typical biomolecules like DNA, protein, peptide, and virus, as well as bio-body like bacteria for the creation of various nanostructures and nanomaterials.

### 2.1. DNA-Based Nanostructures

**Figure 1:**
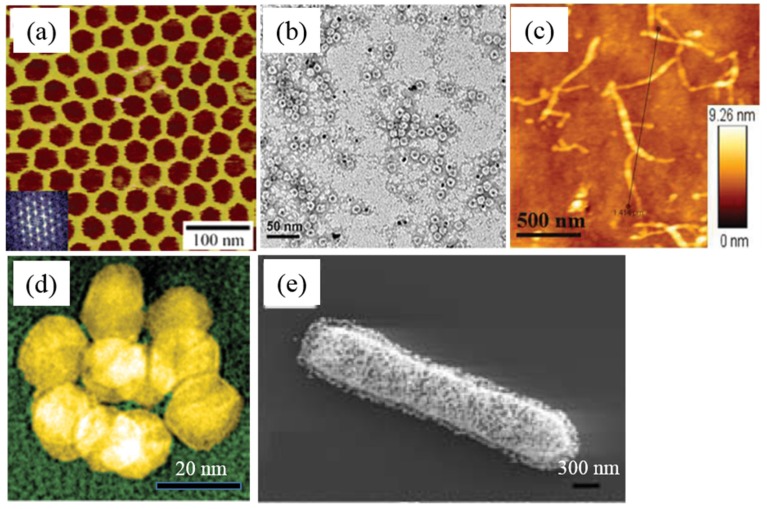
Biomimetic nanostructures: (**a**) DNA 2D structure (Reprinted with permission from [29], published by American Chemical Society, 2005); (**b**) ferritin-based Pt NPs (Reprinted with permission from [31], published by American Chemical Society, 2013); (**c**) peptide nanofiber-graphene quantum dot hybrids (Reprinted with permission from [32], published by WILEY-VCH Verlag GmbH & Co., 2015); (**d**) virus-protein hybrid nanostructures [33]; (**e**) bacterium-based NPs (Reprinted with permission from [34], published by WILEY-VCH Verlag GmbH & Co., 2005).

The synthesis of DNA oligonucleotide and the subsequent development of chemical modification of DNA molecules promote the fabrication and applications of DNA-based nanostructures and nanomaterials. In 1982, the Seeman group for the first time designed self-assembled DNA supra-structures by the molecular recognition of DNA oligonucleotide [25]. After that, they further designed a few branched DNA junctions and then made complex one-, two-, and three-dimensional (1D, 2D, and 3D) nanostructures by selecting other complimentary DNA building blocks to form DNA hybridization [26,27,28]. Their studies paved the way to synthesize DNA-based suprastructure materials. Later, Mao and co-workers reported the creation of porous, hexagonal, and 2D DNA arrays by the self-assembly of three-point-star DNA motifs [29], as shown in Figure 1a. The pores of the formed DNA arrays are hexagons, whose edges are about 18 nm long, and the whole size of the arrays are as large as 1 mM. This kind of DNA 2D materials can be used as the filer membrane for nanoliquid or NPs and substrate for the bioimaging of virus and large protein molecules. In another study, Rothemund demonstrated a bottom-up fabrication of arbitrary 2D DNA nanoscale shapes and patterns by folding long ssDNA molecules [30]. The obtained DNA structures have a diameter of roughly 100 nm and various shapes like squares, disks and five-pointed stars with a spatial resolution of 6 nm.

Other research groups like the Yan group [35,36,37], Fan group [38,39,40], Shih group [41], Yin group [42], and Simmel group [43] have done a lot of corresponding works by designing and assembling DNA building blocks, and various DNA suprastructures have been created. For example, Douglas and co-workers demonstrated the bottom-up design and assembly of DNA nanostructures with different shapes from monolith to square nut, railed bridge, genie bottle, stacked cross, and slotted cross with precisely controlled dimensions ranging from 10 nm to 100 nm [41]. In another typical case, Ke *et al.* described a simple and robust method to construct complex 3D DNA structures by using short synthetic DNA bricks [42]. Several review papers on the bottom-up synthesis and bioapplications of various DNA nanoshapes are recommended if the readers want to get more details [44,45,46,47]. Beside DNA molecules, RNA can also be used for the fabrication of complex structures. Recently, Geary and co-workers for the first time introduced the design of artificial RNA structures by using an architecture that fold from a single strand, in which arrays of antiparallel RNA helices are precisely organized by RNA tertiary motifs and a new type pattern [48]. By this strategy, they created RNA tiles and further assembled the tiles into hexagonal lattices.

The self-assembled DNA supra-structures are the excellent templates for the fabrication of 2D functional materials. For example, Kiehl *et al.* reported the laying of gold NPs onto a preassembled 2D DNA scaffold by using the *in situ* hybridization of DNA-functionalized NPs with the free DNA strand on the scaffold [49]. Aldaye *et al.* reported the creation of the sequential self-assembled DNA hexagon and the further organization of gold NPs on the formed 2D DNA template [50]. In another case, Yan *et al.* presented the self-assembly of protein and silver NPs on the pre-created DNA 2D nanoarrays and 1D nanoribbons, respectively [51]. Recently, Qi and co-workers prepared prescribed structures by using DNA as programmable and sequence-specific glues, and shape-controlled hydrogel units [52]. By using the DNA origami technique, metallic gold NPs can be precisely created on the self-assembled DNA nanostructures [53], which can be utilized as the high-yield production of plasmonic structures. On the other hand, various metallic NPs (gold, silver, and copper) can be synthesized on the self-assembled 2D and 3D DNA origamis as molds [54,55,56]. Beside the self-assembled artificial DNA structures, some natural DNA structures can also be used as templates for the synthesis of 1D and 2D nanostructures and nanomaterials [57,58]. In our previous works, we used plasmid DNA molecules for the preparation of metallic nanowires [59], Cu_2_O nanowires [60], and silver nanorings [61]. In addition, we have created 2D DNA networks and applied the networks for further fabrication of silver nanowires and gold NP film [62,63].

### 2.2. Protein-Based Nanostructures

Protein molecules are also the excellent precursors for preparation of functional nanostructures and nanomaterials. Firstly, protein molecules can be used as biocompatible agents to conjugate with NPs to form stable and functional nanohybrids. For example, the globular protein bovine serum albumin (BSA)-conjugated silver sulfide and gold NPs have been synthesized by the one-step reaction [64,65]. In another study, Rangnekar *et al.* reported the synthesis of α-amylase-modified gold NPs by a green synthesis procedure [66], in which α-amylase served as both the reduction and capping agents for NPs. It is well know that the metallic NPs functionalized with protein or other biomolecules can act as an electron relay between the biocatalyst and the electrode, which can mediate the growth of metallic NPs [67]. Previously, we prepared the lysozyme monolayer-protected gold NPs by the one-step reduction of the mixed solution of HAuCl_4_ and lysozyme [68,69], and we found that the protein-stabilized NPs could self-assembled into network and nanowire structures upon aging under ambient temperature due to the dipole-dipole attraction between NPs.

Secondly, protein molecules can serve as the soft templates for the direct formation of metal and semiconductor nanostructures. Ferritin is a cage-like protein involved in the biomineralization of iron oxide particles. After removing the ferrihydrite from the inner core of ferritin by using acids, the obtained ferritin have shown wide applications for the synthesis of ferritin-inorganic NP nanohybrids with very good dispersity and solubility [70,71]. Recently, Qiu and co-workers reported the ferritin-templated synthesis of Pt NPs (Figure 1b), and further investigated the self-assembly of ferritin-Pt NPs on porous graphene network for the electrocatalysis application [31]. Their study demonstrated that protein nanocage templating and assembly are promising strategies for the fabrication of function nanomaterials for catalysis and fuel cell applications. Very recently, Matsumoto *et al.* showed that a high-throughput protein selection technique can be applied to enhance intracellular molecular-level biomineralization within ferritin variants, resulting in proteins with the ability to induce magnetic phenotypes [72]. The engineering ferritin can influence the magnetic resonance imaging signals at multiple scales and serve as building blocks for intracellular magnetic devices. In our previous studies, we investigated the conjugation and assembly of ferritin molecules along carbon nanotubes (CNTs) and graphene nanosheets, and further synthesized functional CNT-FePt and graphene-FePt nanohybrids by using the ferritin template [73,74]. In addition, a few globular proteins, such as fibrinogen, globulin, hemoglobin, and fibronectin showed the great potentials for the synthesis of protein-based NPs [75,76]. Another linear protein, collagen (about 280 nm), has also been used for the nucleation and growth of gold and silver NPs previously in our group [77,78].

Protein-DNA hybrid assemblies can also be fabricated by assembling protein molecules onto the programmed DNA suprastructures [39,79,80,81]. For instance, Fu and co-workers reported the assembly of glucose oxidase/horseradish peroxidase enzyme pairs on the created DNA origami tiles with controlled interenzyme spacing and position [79]. The distance between enzymes was adjusted from 10 nm to 65 nm. They found that the distance between enzymes is responsible for the activity of enzymes. In a further study, they demonstrated that a DNA nanostructure can be used to create a multi-enzyme complex in which an artificial swinging arm facilitates hydride transfer between two coupled dehydrogenases [80]. In another case, Linko *et al.* presented a nanoscale reactor assembled from tuneable and spatially addressable tubular DNA origami units by assembling glucose oxidase (GOx)/horseradish peroxidase enzyme pairs [81]. Their study indicate that the fabricated reactor could be used as a nanoscale diagnostic tool.

Besides the natural proteins, the self-assembled supra-structures like protein nanofibers have been also widely utilized for the fabrication of various nanomaterials. A direct and feasible strategy to create protein nanofibers is by the self-assembly of proteins. There are several methods that may initiate the conformation transition and self-assembly of protein molecules, such as low pH value, temperature, metal ions concentration, and denaturants. For example, Krebs *et al.* reported the formation of amyloid nanofibers from wild-type hen lysozyme at pH 2.0 [82]. Arnaudov *et al.* studied the effects of pH and temperature on nanofibers formation from hen egg white lysozyme [83]. They found that a low pH and temperatures close to the midpoint temperature for protein unfolding promote the formation of nanofibers. Akkermans *et al.* also reported the formation of β-lactoglobulin nanofibers by incubating protein solutions in pH 2.0 at 80 °C for approximately 20 h [84]. The above studies suggested that the formation of protein nanofibers was affected by pH and metal ions concentration in the solution. Protein nanofibers can also be created by introducing several protein denaturants, such as ethanol [84,85], trifluoroethanol [86], and sodium dodecyl sulfate [87] into protein solution. Previously, we investigated the formation of fibrinogen and fibronetin nanofibers in ethanol and acidic solutions by controlling the concentration of proteins and ethanol, the pH value of system, the temperature, as well as the incubation time [88,89,90,91]. All these studies indicated that self-assembly is a fundamental phenomenon for proteins, and it is also the predominant method for the formation of protein nanofibers.

### 2.3. Peptide-Based Nanostructures

Peptide is another type of molecular building block that can be widely used for the creation of functional nanostructures and nanomaterials. Previous studies indicate that the self-assembly of peptide molecules to different nanostructures and the physical and chemical properties of the created peptide nanostructures can be controlled by designing the sequence of amino acids of peptide molecules [20,92]. In addition, the unique properties of peptide nanostructures make it possible to fabricate various nanodevices by using the self-assembled peptide nanostructures as building blocks. Firstly, the designed peptide nanofibers or nanotubes have novel functions like molecular recognition and biomimetic mineralization or metallization, which can be benefit to the fabrication of nanodivices and biosensors [93]. Secondly, the functional motifs or groups can be easily added into the desired position of peptide molecules by chemical synthesis, which is very important for the further modification of peptide nanostructures with other nanomaterials like NPs [94]. Thirdly, the synthesis of peptide is simple, and the self-assembled peptide nanostructures are very stable even at high temperature, which is suitable for the high-temperature fabrication procedures of nanodevices.

Peptide molecules have the similar ability as protein for the biomimetic synthesis of metallic NPs. For example, Naik and co-workers reported the *in vitro* biosynthesis of silver NPs using the silver-binding peptides identified form a combinatorial phage display peptide library, and they found that the peptide molecules can accelerate the nucleation of metal clusters and the selected peptides can interact with these clusters to further promote the growth of particular phases [95]. In a further study, they developed a simple, one-pot process for synthesizing monodisperse gold NPs in aqueous solution using multifunctional peptides. They utilized the peptide contains a gold binding domain and biomolecular recognition domain, and therefore the designed peptide has the abilities not only reduce choloroaurate ions and coat the formed gold NPs, but also recognize with specific antibodies [96]. In another case, Graf *et al.* reported the synthesis, surface chemistry, and self-assembly of the peptide-coated silver NPs [97], and they found that the designed peptide can not only control the colloidal properties, but also influence the crystal structure of the individual NPs. In addition, they suggested the created peptide-silver hybrid nanostructures can be used as building blocks for the construction of new meta-materials with tunable properties. To understand the synthesis mechanism of metal NPs mediated by peptides, a lot of theoretical studies have been done [98,99].

Another important peptide supra-structure is the nanofibers or nanotubes formed by the self-assembly of peptide molecules. Some design rules allow peptides to be used as building blocks for self-assembly to form fibrous peptide nanostructures [20]. There are two kinds of design rules that can be followed to create self-assembled peptide nanofiber. The first category utilizes the basic conformational units of naturally existing proteins, β-sheets and turns, α-helices and coiled coils. β-sheets are well known for their ability to assemble into long fibrous structures, as is seen in amyloid diseases, such as Alzheimer’s and Parkinson’s diseases. Some peptide sequences were thought to be critical for the formation of amyloid nanofibers and cause of diseases. The most studied peptide sequences are the amyloid-β peptide with 39–43 amino acids, and many fibrous structures have been created by controlled assembly. For example, Ray *et al.* reported that a water-soluble tripeptide (Val-Ile-Ala, VIA) that originated from the Aβ_40–42_ can self-assemble into amyloid-like nanofibers [100]. Inspired by the rules provided by nature, scientists started to create new soft materials that are not harmful to humans by designing the amino acid sequence. Zhang *et al.* first demonstrated the use of β-sheets for the design of new fibrous materials in the early 1990s [101]. Their work indicated that it was possible to create a fibrous peptide structure containing β-sheets by creating a pattern of hydrophobic amino acids and complementary charges between peptides, such as the pattern of (AEAEAKAK)_2_. Similar peptide patterns, such as (RADA)_4_, (RARADADA)_2_,and (KLDL)_3_, have been used to create peptide nanofibers [102,103,104]. An alternative to using β-sheets as a basis for a fibrous nanostructure are α-helices, and one example of a well-studied and well-defined α helical motif is the coiled coil [105]. An extensively studied design of a fibrous coiled-coil based system has been demonstrated by the Woolfson group using the “sticky ends” assembly [106]. In this system, they described two peptides that are designed to combine in an offset manner to give a “sticky end” heterodimeric leucine zipper, which promotes longitudinal assembly of the fibers, and the prepared nanofibers tend to be tens of microns long and tens of nanometers thick.

The second categorical rules for the creation of peptide nanofibers are based on the peptide derivatives, for instance, peptide amphiphile (PA) and π-stacking systems. PA consists of oligo-peptides that are modified with a hydrophobic alkyl tail to form molecules with distinctly hydrophobic and hydrophilic ends, similar to lipids. This kind of PAs has a tendency to self-organize in aqueous solution so that hydrophobic domains are buried away from water while hydrophilic regions are exposed to water. In this way the PA can self-assemble into various types of micelles and vesicles. In 2001, Stupp’s group first reported the preparation of PA with mono-alkyl chains attached via the N-termini [107]. The peptide contained no proline residues and can self-assemble into fibrous cylindrical micelles in which the peptide portion adopted a largely β-sheet character. Their work indicated that the self-assembly of PA can be controlled by simple adjustment of pH of PA solution. Later studies showed that PA nanofibers could be self-assembled at low pH or high pH depending on the sequence selection [108,109]. π-stacking is also a special way of self-assembly of peptide derivatives. For example, Reches *et al.* demonstrated that amyloid peptides with a core sequence of di-phenylalanine can self-assemble to form stable peptide nanotubes [110].

The self-assembled peptide nanofibers and nanotubes are excellent templates for the creation of metallic nanowires. Previous studies indicate that it is possible to coat metal NPs on the formed peptide nanofibers or nanotubes by designing the peptide molecules with specific binding motifs toward metal NPs [110,111,112]. The uniform, high density, and high-crystalline coating of metal NPs on the surface or inner of nanofibers or nanotubes can be obtained by chemical reduction of the specific bound metal ions. Recently, we have performed a few studies on the design of peptide molecules with specific motifs for the creation of functional peptide nanofibers [32,113,114]. For instance, we designed a peptide molecule with two functional motifs, which relate to the binding of silver NPs and the formation of nanofibers [113]. The created nanofiber-based silver nanowires have potential application for the fabrication of electrochemical biosensors. In the further work, Su *et al.* designed another peptide molecule with three functional motifs, which are responsible for the self-assembly, binding with graphene quantum dots, and specific targeting with cancer cells [32], and the created nanofiber-quantum dot nanohybrids were shown in Figure 1c. Their results indicate that the functional nanofiber-quantum dot nanohybrid has potential application for simulateous targeting and labelling cancer cells.

### 2.4. Virus-Based Nanostructures

The protein shells of some virus are protein supra-structures. For example, the *Poliovirus* is a sphere with a diameter of 30 nm, the tobacco mosaic virus (TMV) is a rod-like structure with a diameter of 18 nm and a length of 300 nm, and Cowpea chlorotic mottle virus (CCMV) is an assembled capsid with complex structures. All these virus structures with specific size, shape, and properties can be used as the potential precursors for the synthesis of functional nanomaterials [115].

TMV is the most used candidate for the synthesis of inorganic nanomaterials. There are many amino acids like Glu, Asp, Arg, and Lys in TMV, which are benefit for the chemical modification, mineralization, and metallization to form inorganic nanostructures [116]. In 1999, Shenton and co-workers for the first time reported the synthesis of inorganic-organic nanotube composites by the templated mineralization of TMV [117]. With the TMV template, they prepared SiO_2_, PbS, CdS, and Fe_2_O_3_ nanotubes by adjusting the pH value of the reaction system to 2.5, 5, 7, and 9, respectively. In the next years, many kind of nanomaterials based on TMV, such as silver, platinum, nickel, cobalt, ZnO, and polymer nanowires, have been synthesized [118,119,120,121,122].

CCMV is also another virus that can be used for the synthesis of inorganic nanomaterials. For instance, Douglas *et al.* reported the mineralization of two polyoxometalate species, paratungstate and decavanadata, and the encapsulation of an anionic polymer inside CCMV could be achieved by controlling the pH-dependent gating of the virion’s pores [123]. They suggested that the diversity in size and shape of CCMV particles make this method a versatile strategy for material synthesis and molecular entrapment. Recently, Mikkilä and co-workers showed how DNA origamis can be coated with CCMV in order to facilitate efficient cell transfection [124]. To achieve this aim, they used a DNA origami (71 nm × 92 nm) as template to assemble CCMV. Their study indicates that the ability of DNA-CCMV complexes to bind and transfect human cells was 13 times higher compared to the pure DNA origami structures. Besides TMV and CCMV, some other virus, like M13 bacteriophage [125] and cowpea mosaic virus (CPMV) [33] can also be utilized for the synthesis of virus-based nanomaterials. For example, recently Fontana and co-workers demonstrated a self-assembly strategy to create three-dimensional, isosahedral plasmonic nanoclusters by using the genetically engineered CPMV template [33]. They found that the gold nanocluster can be covalently attached onto the surface of CPMV at predefined locations to form icosahedral symmetry between the nanoclusters, as shown in Figure 1d. Please study the review paper on the synthesis and application of virus-based hybrid nanomaterials to get more details [126].

### 2.5. Microorganism-Based Nanostructures

Bacterium is one of the microorganisms existed in the nature. There are about 5 × 10^3^ bacteria on our Earth, which form a biomass larger than that of all plants and animals. Typically, bacteria have a size of a few μm and a number of shapes from sphere to rod and spiral. Therefore, bacteria are the potential templates for the biomimetic synthesis of functional nanomaterials. For example, Davis *et al.* for the first time utilized a bacterial supra-structure consisting of a thread of coaligned multicellular filaments of *Bacillus subtilis* to produce ordered macroporous fibers of either amorphous silica or ordered mesoporous silica [127]. The formed mesoporous silica framework has 0.5 μm wide channels with curved walls of either silica or mesoporous silica about 50–200 nm thickness. This kind of mesoporous materials have potential applications in catalysis, molecular separation, and biomaterial engineering. Later, Klaus and co-workers reported the biosynthesis of silver-based single crystals with well-defined compositions and shapes by using the bacterium of *Pseudomonas stutzeri* AG 259 [128]. The bacteria were separated from the silver minerals and incubated with highly concentrated AgNO_3_ solution at 30 °C for 48 h in the dark to get the triangle and hexagon silver single crystals. The synthesized silver-based single crystals are embedded in the organic matrix of the bacteria.

Inspired by these studies, different species of bacterial have been used for the synthesis of bacterium-based metal hybrid nanomaterials [34,129,130,131,132]. For instance, Berry *et al.* reported a self-assembly strategy to build hybrid devices that use the biological response of a microorganism to control the electrical properties of the system [34]. In this system, a monolayer of gold NPs was coated onto the peptidoglycan membrane of a live Gram-positive bacterium, as shown in Figure 1e. The electrical properties of the fabricated bioelectronics device can be controlled by actuating the peptidoglycan layer of the bacterium. This study paves the way to obtain active coupling between microorganisms and the electrical, optical, and magnetic nanodevices. In another case, Shankar and co-workers reported the rapid synthesis of gold, silver, and gold-silver core-shell NPs by using Neem (*Azadirachta indic*) leaf broth [129].

### 2.6. Synthesis Strategies of Biomimetic Hybrid Nanomaterials

Based on the above introduction on the preparation of biomolecule/biobody-based nanostructures, it can be understood that DNA, protein, peptide, virus and bacterium show powerful ability to create functional hybrid nanomaterials by interacting with other building blocks like NPs, quantum dots, carbon nanotubes, and graphene nanosheets. Based on the biomolecular templates or the self-assembled biomolecular supra-structures, many different bio-hybrids like zero dimensional (0D), one dimensional (1D), two dimensional (2D), and three dimensional (3D) hybrid nanomaterials can be prepared effectively.

Usually, there are three typical strategies can be utilized to achieve this aim. Firstly, metallic ions are electrostatic adsorbed onto the as-formed bionanostructures and then the bio-hybrids (like biomolecule-NP) can be created by the simple chemical reduction [31,60,61,62,63,64,65,66,67,68,69,70,71,72,73,75,77,88,113]. This method is simple and direct, and the size of metallic or semiconductor NPs on the bionanostructures can be controlled by adjusting the concentration of metallic ions and reduction reagent, as well as the pH value of system. Secondly, the bio-hybrids can also be created by mixing the biomolecules or supra-structure with metallic ions by the biomimetic mineralization and metallization [76,89,91,114]. The specific biomolecules used for this method should have the unique ability to mediate the adsorption of metallic ions and promote the formation of NPs or microscale nanostructures. Compared to the first strategy, this strategy is much simple and green, but the size and morphology of the biomimetic nanostructure are harder to control and the synthesis period is longer. Thirdly, it is possible to fabricate the bio-hybrids by assembling the as-prepared NPs to the bionanostructures by covalent and electrostatic interactions [32,49,50,51,57,59,62,90]. It is the mostly used strategy for the synthesis of biomolecule-based nanostructures. There are enough functional groups on the surface of biomolecules, which are benefit for the covalent reaction with modified NPs. On the other hand, the created biomimetic nanostructures can be transferred from positive to negative by adjusting the pH value of the system to mediate the electrostatic interactions with NPs. To make it more clear, here we present a table to classify the various synthesis strategies for the biomolecule- and microorganism-based hybrid nanomaterials, as shown in Table 1.

**Table 1:** Synthesis strategies for the biomimetic hybrid nanomaterials.

Bionanostructures	Hybrids	Strategy	Advantages	Ref.
0D	protein-NP	chemical reduction of adsorbed metal ions on/in biomolecules, or biomimetic synthesis	simple, size-controllable, economic, soluble	[64,65,66,67,77,78]
	peptide-NP			[93,94]
	virus-NP			[33]
	bacterium-NP			[127,128,129]
1D	DNA-NP	chemical reduction of adsorbed ions or direct self-assembly of as-prepared NPs	nanowire synthesis, simple, size-controllable	[58,59,60,61]
	protein-CNT-NP			[73]
	nanofiber-NP			[88,89,90]
	virus-NP			[117,118,119,120,121,122,125]
	bacterium-NP			[34,129,130,131,132]
2D	DNA grid-NP	chemical reduction of adsorbed ions or direct self-assembly of as-prepared NPs	2D scaffold synthesis, green synthesis, size-controllable, function adjustable	[49,50]
	DNA array-NP			[51]
	DNA network-NP			[62,63]
	protein-graphene-NP			[72,74]
	nanofiber-graphene-NP			[32,113,114]
3D	DNA-NP	biomimetic mineralization	green synthesis, controllable	[54,55,56]
	nanofiber scaffold-NP			[91]
	virus-NP			[33]

## 3. Sensor Applications of Biomimetic Nanostructures

**Figure 2:**
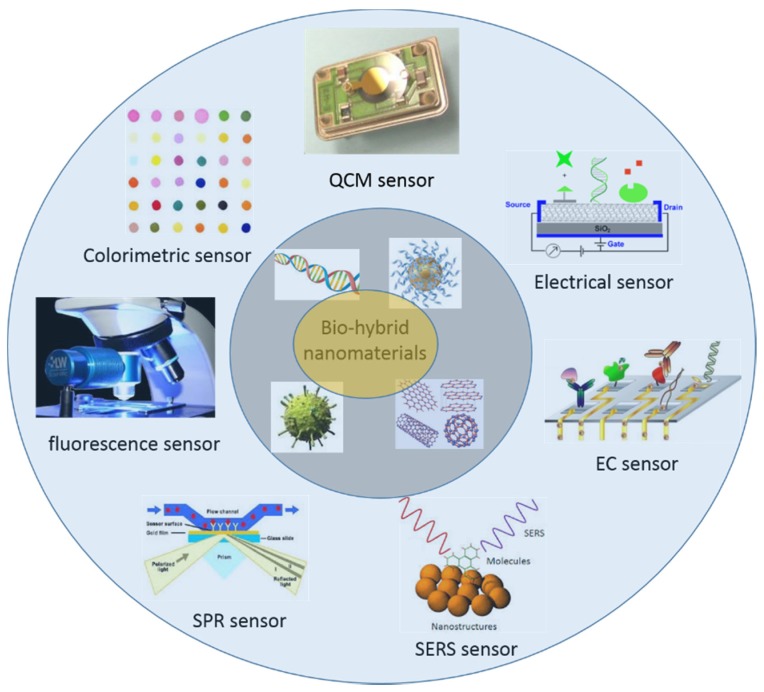
Potential sensor applications of the biomimetic hybrid nanomaterials.

The development of nanotechnology greatly improves the applications of biomolecule-based nanostructures in biosensors and biomedical engineering [133]. Many functional NPs and other nanoscale building blocks have been conjugated onto biomolecules and biomolecular supra-structures for the promising applications in biosensors, bio-diagnosis, and bio-therapy. Compared to the traditional sensing techniques based on molecular probes, the biosensors and biodetection techniques fabricated with the biomolecule-based nanostructures show better selectivity and higher sensitivity. In this part, we would like to present the advances in the sensor applications of biomimetic nanostructures and nanomaterials for colorimetric, fluorescence, SPR, SERS, EC, electrical, and QCM detection, as shown in Figure 2.

### 3.1. Colorimetric Sensing

Colorimetric sensors are extremely attractive with the development of sensing techniques because the detection with acceptable resolution can be easily read out with the naked eye by this technique. In some cases, those simple colorimetric sensors can even eliminate the use of analytical instruments. The most widely used colorimetric sensor is the metallic NP-based scanometric method developed by Mirkin and co-workers [134,135,136]. In these cases, gold NPs were first modified with a probe DNA or an aptamer, and the adding of analytes into the NP system can promote the aggregation of gold NPs by the DNA hybridization or aptamer-based molecular recognition, causing the obvious color change of the nanoparticle solution. The combination of DNAzyme and gold NPs can also utilized for the colorimetric sensing of metallic ions. For example, Lu and co-workers for the first time reported the fabrication of colorimetric sensors of Pb^2+^, Hg^2+^, cocaine, and adenosine by using the DNAzyme-directed assembly of gold NPs [137,138,139,140]. The DNAzyme-gold NP sensors created by the DNAzyme directed assembly are highly sensitive and selective for the analytes.

Protein-mimetic metallic NPs can also be used for the colorimetric sensing [141,142]. In a typical example, Li *et al.* reported the biomimetic synthesis of Pt NPs with the average diameter of 2.0 nm by using bovine serum albumin (BSA) as the nucleation template [142]. The synthesized BSA-Pt hybrid nanozymes possess highly peroxidase-like activity toward 3,3’,5,5’-tetramethylbenzidine (TMB) and hydrogen peroxide (H_2_O_2_). They found that Hg^2+^ can down-regulate the enzymatic activity of Pt NPs and cause the color change from dark blue to light blue. This technique can provide a direct and simple detection of Hg^2+^ with a limitation detection of about 7.2 nm and a linear response range of 0–120 nm. The fabricated colorimetric sensor can be potentially applied for the quantitative determination of Hg^2+^ ions in aqueous solution and drinking water with very good selectivity and high sensitivity.

**Figure 3:**
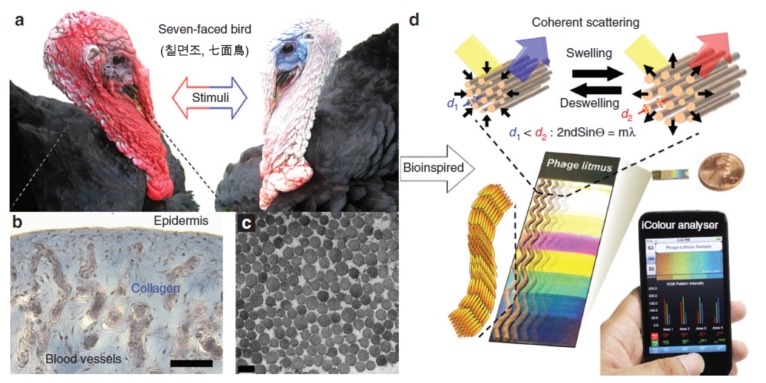
Biomimetic M13 phage-based colorimetric sensors. (**a**) Color changes of turkey skin when they get flustered; (**b**,**c**) Structural characterizations of turkey skin; (**d**) Bioinspired M13 phage arrays for colorimetric sensing. (Reprinted with permission from [143], published by Nature Publishing Group, 2014).

Many materials in nature change colors in response to stimuli, making them attractive for use as the colorimetric sensing platform. In a very interesting work presented by Oh *et al.* recently [143], biomimetic virus-based colorimetric biosensors were fabricated by using the genetically engineered M13 phage, as shown in Figure 3. The detection mechanism mimics the color change of turkey skins from red to white or blue when excited (Figure 3a). The experimental characterizations proved that formation of blue of turkey skin is corresponding to the coherent scattering of light from collagen bundle-based nanostructures (Figure 3b,c). Inspired by this phenomenon, Oh and co-workers fabricated the tunable M13 phage-based arrays of differently colored phage litmus, as shown in Figure 3d. After putting the phage arrays into external chemical, the matrices will swell or shrink rapidly and result in color changes similar to those seen on turkeys when they get flustered. To improve the selectivity of this colorimetric sensor, a trinitrotoluene (TNT)-binding peptide was incorporated onto the phage arrays. They experiments indicate that the detection limit of TNT with the TNT-binding phage litmus sensor can be down to 300 ppb with the common smart device like iPhone. The strategies shown in this work suggest that virus-based colorimetric biosensor has great potentials for the easy detection of other harmful toxicants and pathogens by using the specific binding motifs.

### 3.2. Fluorescence Sensing

**Figure 4:**
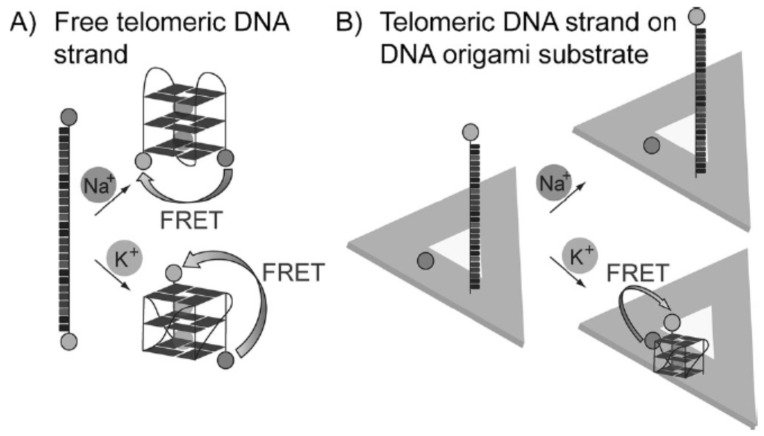
Selective detection of K^+^ with telomeric DNA strand and DNA origami.（**A**） Free telomeric DNA strand; (**B**) Telomeric DNA strand on DNA origami substrate (Reprinted with permission from [146], published by WILEY-VCH Verlag GmbH & Co., 2014).

Biomolecule-based nanostructures can also be employed for the fluorescence sensing. A typical example is the DNA origami structures [144]. Previous studies indicate that the 2D DNA origami structures can be used as a platform to detect RNA and metallic ions like Na^+^ and K^+^ [145,146]. For example, recently Olejko *et al.* used Förster resonance energy transfer (FRET) to investigate in detail the Na^+^ and K^+^ induced folding of G-quadruplexes from both free telomere sequences and telomere sequences attached to the triangular DNA origami platforms [146], as shown in Figure 4. They found that the free telomeric DNA strand can be transferred to G-quadruplexes in the presence of Na^+^ and K^+^ (Figure 4A), but when the telomeric DNA strand was bound onto the DNA origami structure only the K^+^ can promote the formation of G-quadruplexes for FRET. This technique can be applied for selective detection of K^+^ from 0.5 to 50 mM even in the presence of high Na^+^ concentration.

Fluorescence-labelled DNA-graphene nanohybrids can also be used as FRET sensing platforms for biomolecules. For instance, Lu *et al.* for the first time utilized a fluorescence-labelled ssDNA molecule to bind with graphene oxide nanosheets, and they found that graphene oxide can quench the fluorescence of the dye, but when a target was added into the system the specific binding between the dye-labelled DNA and target molecules will release the DNA molecules from the graphene oxide surface and result in restoration of dye fluorescence [147]. This technique can be used for sensing DNA and protein molecules. In another case, Chang and co-workers applied the similar strategy for the detection of thrombin [148], and a detection limit as low as 31.3 pM was obtained based on the graphene FRET aptasensor. Recently, Zhang *et al.* reported the preparation of dual-purpose fluorescence and electrochemical biosensor platform based on polymer nanofibers and graphene quantum dots. By conjugating the GOx enzyme onto the hybrid nanofibers, the fabricated fluorescent biosensor reveals high sensitivity and selectivity toward the detection of glucose and H_2_O_2_ by measuring the changes of fluorescence intensity [149].

Protein-metal NP hybrids have also been used for the fabrication of fluorescence sensors. Previously, Xie and co-workers found that BSA can be uses as a temple for the formation of gold nanoclusters with strong fluorescence, and the addition of Hg^2+^ into the gold nanocluster solution can cause the quench of fluorescence [150]. Based on this finding, they designed a simple label-free method for the selective and sensitive detection of Hg^2+^ within the linear range of 1–20 nM. In another study, the BSA-mimetic silver nanoclusters were also used for the label-free fluorescent detection of Hg^2+^ [151].

### 3.3. SPR Sensing

SPR is a physical optics phenomenon and it uses an optical method to measure a change in refractive index of the medium in close vicinity of a metal surface [152]. SPR biosensor has received a great of research interests due to its advantages than other optical biosensors, such as label-free, real-time, fast response and multiscale analysis at one time [153].

Previous studies indicate that the coupling of biomolecules and gold NPs can greatly amplify the SPR shifts, and therefore the biomolecule-gold NP hybrid systems have been widely used for label-free detection of DNA, protein, and metallic ions [154,155,156]. For example, Pelossof *et al.* reported the SPR biosensors for amplified detection of DNA, adenosine, and Hg^2+^ by using the hemin/G-quadruplexes and gold NPs [154]. Firstly, they synthesized the three types of gold NPs modified with hemin/G-quadruplexes by using the specific ssDNA aptamers for sensing DNA, adenosine, Hg^2+^. Secondly, the created gold NPs were assembled onto the thiol-modified gold surface. After the adding of targets into the system, the targets will interact with the corresponding aptamer to form a hairpin nanostructure. By this way, a functional gold NP monolayer-modified surface was fabricated. The resulting dielectric changes on the surface exhibited shifts in the obtained SPR spectra, making it possible to get the read-out signals for the molecular recognition. In another case, Bai and co-workers reported the fabrication of aptamer/thrombin/aptamer-gold NP sandwich structure, and further utilized the created SPR biosensor for label-free detection of thrombin [155]. The obtained SPR signal has a good linear relationship with thrombin concentration in the range of 0.1–75 nm, and the detection limit for thrombin with the fabricated SPR biosensor was determined to be as low as 0.1 nM.

**Figure 5:**
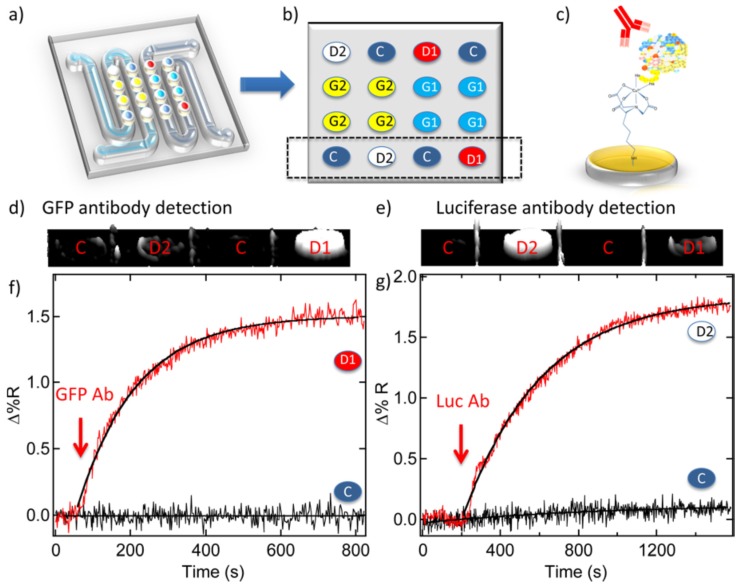
DNA-based protein microarrays for SPR imaging biosensor application (**a**) Five-component DNA microarray; (**b**) Spatial diagram of the five components on the DNA microarray; (**c**) Schematic binding of antibody onto protein microarray; (**d,e**) SPR imaging taken before and after binding the antibody to (**d**) D1 and (**e**) D2; (**f**) Real-time SPR imaging adsorption kinetics of anti-GFP binding onto D1; (**g**) The similar adsorption kinetics of anti-luciferase binding onto D2. (Reprinted with permission from [157], published by American Chemical Society, 2012).

Recently, Seefeld *et al.* reported a very interesting work on the biosensing of antibody by using the developed SPR imaging technique [157]. In their work, they utilized a generator with DNA microarrays to on-chip synthesis of protein microarrays on the detector. The single stranded mRNA molecules are transcribed from the surface-bound dsDNA on the generator and the translated protein molecules are captured by the detector for the SPR imaging biosensing. Figure 5 shows the application of the synthesized green fluorescent protein (GFP)-luciferase protein microarrays for the detection of the anti-GFP and anti-luciferase antibodies. They used a five-component, 16-element DNA microarray chip (Figure 5a) to synthesize protein microarray for biosensing, in which five components are labelled as 4 GFP generator elements (G1), 4 luciferase generator elements (G2), 2 GFP detector elements (D1), 2 luciferase detector elements (D2), and 4 control elements (C), respectively, as shown in Figure 5b. The fabricated microarray was then applied for the SPR imaging antibody biosensing (Figure 5c). The SPR imaging difference images taken before and after the incubation in anti-GFP and anti-luciferase solutions (50 nM and 100 nM, respectively) indicated that the adsorption of antibodies can be clearly observed in these images as the detector D1 and D2 show significant signals (Figure 5d,e). The corresponding real-time SPR imaging adsorption kinetics measurements further identify the strong adsorption of anti-GFP and anti-luciferase towards the relative detectors (Figure 5f,g). This novel and convenient strategy for the fabrication of the DNA-based on-chip protein microarray will have potential applications for the multiple biosensing in clinical and research fields.

### 3.4. SERS Sensing

**Figure 6:**
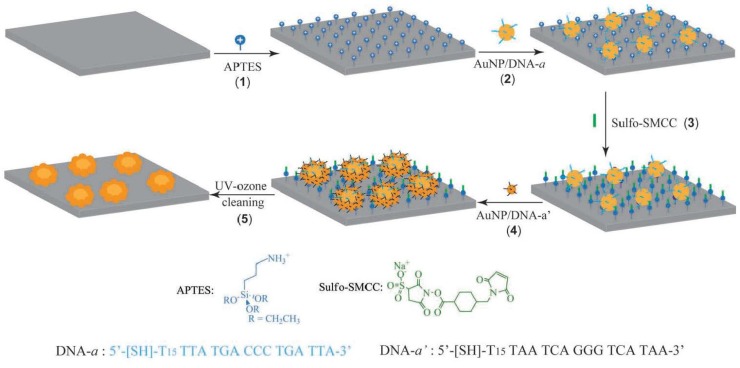
Schematic presentation for the fabrication of core-satellite near-IR SERS sensor (Reprinted with permission from [159], published by WILEY-VCH Verlag GmbH & Co., 2012).

SERS has been shown to be a useful tool for the detection of low-concentration analytes, sometimes even achieving single-molecule sensitivity. One of the important fields of SERS study is how to prepare SERS-active substrates that can provide great sensitivity and good reproducibility. Biomolecule-based metal nanostructures are the excellent substrates for the SERS detection. We have made the first explorations by applying the 2D DNA network decorated with silver NPs for the highly sensitive SERS detection of R6G and 4-aminothiophenol [62]. We found that the DNA network provides an excellent porous template for the self-assembly of silver NPs to form SERS-active substrate with high activity and signal-to-noise. In another study, we fabricated a highly active SERS substrate by using the layer-by-layer assembly of *λ*-DNA networks and positive charged silver NPs [158]. Recently, Zheng and co-workers reported a very interesting work on the DNA-directed self-assembly of core-satellite plasmonic nanostructures for the highly sensitive and reproducible near-IR SERS sensor [159]. Figure 6 shows the schematic preparation of the hierarchical self-assembled core-satellite plasmonic nanostructures. Firstly, a glass substrate was modified with 3-aminopropyltriethoxysilane (APTES), and then the negative charged 30 nm gold-DNA NPs can electrostatic immobilized onto the APTES-modified glass. After neutralizing the surface-confined amino groups by the formation of covalent amide bonds with sulfosuccinimidyl-4-[*N*-maleimidomethyl]cyclohexane-1-carboxylate (sulfo-SMCC), another 20 nm gold-DNA NPs (with complimentary ssDNA) can be conjugated onto the 30 nm gold NPs by DNA hybridization. Finally, the surface-confined molecules were removed through UV-ozone cleaning to yield SERS-active surface. This near-IR SERS substrate is highly active and the detection limit is down to single molecular level. In addition, this strategy for creating SERS substrate is relative economic. As for the DNA-based nanostructures for SERS applications, it is recommended to study the previous review paper by Sun *et al.* [160].

Besides the DNA-based nanostructures, protein-based nanostructurs have also been found to have potential applications for SERS detection. For example, we demonstrated that the protein like linear collagen can be used as template to synthesize gold and silver NP linear structures [77,78]. Recently, Wang *et al.* reported that the self-assembled fibronectin nanofibers can also be used for the biomimetic synthesis of silver nanostructures for the SERS detection of 4-aminothiophenol and 2-thiouracil with high sensitivity [161].

Bacteria template can also be used for the biomimetic synthesis of metallic nanostructures for SERS application. Previously, a typical study has been performed by Yang *et al.* [162], who utilized bacteria *cocci* (Gram positive bacteria) as templates to synthesize micron-sized hollow silver microspheres by a simple and cost-effective approach. The synthesized silver microspheres not only have uniform size but also possess hollow and porous structures. The further SERS experiments indicated that the silver microsphere-based SERS substrate has very strong activity toward 2-mercaptopyridine and the detection limit can be as low as 1 × 10^−15^ M with an enhancement factor of 10^11^. Recently, Sun and co-workers reported the *Cacumen Platycladi* leaf extract can be used for the bio-templated synthesis of flower-shaped Au-Pd and Au@Pd NPs for highly active SERS substrates [163,164].

### 3.5. EC Sensing

3D DNA nanostructures are the excellent platforms for the fabrication of EC biosensors. The Fan group in Chinese Academy of Sciences has made great contributions on the fabrication and EC biosensor applications of tetrahedral DNA nanostructures [165,166,167,168,169].

**Figure 7:**
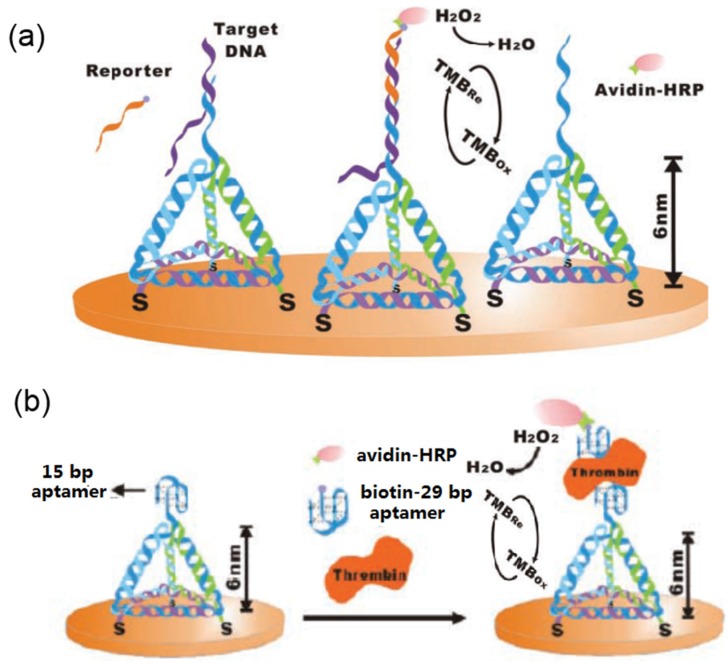
3D tetrahedral DNA nanostructures for EC biosensing of (**a**) DNA and (**b**) thrombin (Reprinted with permission from [165], published by WILEY-VCH Verlag GmbH & Co., 2010).

In a typical study, they designed a DNA tetrahedron nanostructure (shown in Figure 7a) with pendant probe DNA at one vertex and three thiol groups at the other three vertices [165], which can be conjugated onto the surface of gold electrode by three thiol groups but leaving a free-standing probe at the top. The created pyramid-like DNA tetrahedral nanostructures have good mechanical rigidity and structural stability, making them excellent platforms to anchor biomolecules on the surface. In the next step, they further utilized the fabricated 3D tetrahedral DNA nanostructures for the EC sensing of DNA and thrombin molecules, as shown in Figure 7a,b. In the case for sensing DNA, the DNA hybridization between the probe DNA, target DNA, and reporter promotes the formation of dsDNA structure on the top of the tetrahedral DNA nanostructure, which can specific interact with avidin-HRP (horseradish peroxidase), as shown in the middle model of Figure 7a. The presence of target DNA with nM concentration led to a significant increase in amperometic signals corresponding to the catalytic reduction of H_2_O_2_ to H_2_O, but no significant signals for the non-complimentary DNA sequences. This sensing platform can provide a detection limit of 1 pM for target DNA with high sensitivity and selectivity. In the case for sensing thrombin, they replaced the probe DNA with a 15 bp anti-thrombin aptamer. After the addition of thrombin, biotin-aptamer, and avidin-HRP onto the 3D DNA nanostructure, a sandwich-like structure was formed on the top of tetrahedral DNA nanostructure, as shown in Figure 7b. The electrochemical catalysis reduction of H_2_O_2_ to H_2_O can also improve the amperometic signals. This sensor platform exhibits excellent sensitivity toward thrombin with a detection limit of 100 pM. In the next studies, they further utilized the 3D tetrahedral DNA nanostructures for the fabrication of EC biosensors for detecting cocaine [166], antibody [167], and microRNA [168,169], and these DNA tetrahedral structure-based biosensors show improved performances than the DNA hybridization and enzyme immobilization based EC biosensor [170].

Biomacromolecules and their assemblies have the unique ability for biomimetic promotion of the formation of novel and functional nanomaterials. Protein molecules are the potential templates for the synthesis of metal NPs, which can be utilized for the modification of electrode for the electrochemical sensing. For example, the BSA-templated silver and gold NPs have been employed for the fabrication of label-free retinol-binding protein and tumor cell sensors [171,172]. In order to improve the electrochemical signals, protein-templated metal NPs were further conjugated with CNTs and graphene nanosheets for the modification of electrodes. For example, previously we synthesized the hybrid nanomaterials of protein-Pt/CNT [173], ferritin-FePt/CNT [73], ferritin-FePt/graphene [74], and collagen-silver nanowire/graphene [174] for the fabrication of EC glucose and H_2_O_2_ sensors. Recently, Wang *et al.* reported the synthesis of peptide nanofiber-biomimetic silver nanowires on graphene nanosheets, and they further fabricated a high-performance EC H_2_O_2_ sensor based on the created graphene/peptide nanofiber/silver nanowire hybrid materials [113]. The artificial peptide nanofibers were created with a special designed peptide molecule that contains complex motif sequences related to the self-assembly formation of nanofibers and the biomimetic formation of silver NPs. The fabricated EC sensor shows high sensitivity and selectivity, low detection limit, and wide linear range for the determination of H_2_O_2_. This peptide-inspired synthesis of metal nanostructures provides a new idea for the fabrication of functional nanomaterials.

### 3.6. Electrical Sensing

The change of the electrical signals before and after adding analytes into a nanodevice can also be used for label-free sensing. Previously, Park and co-workers reported the fabrication of an array-based electrical detection of DNA with NP probes [175]. In their experiment, microelectrodes with 20 μm gaps were prepared by the standard photolithography on a Si wafer with a 1 µm coating of SiO_2_. They first conjugated some shorter “capture” ssDNA molecules between the two fixed microelectrode and then added gold NPs functionalized with longer “probe” ssDNA and “target” ssDNA into the system. The DNA hybridization between the target DNA and both capture and probe DNA filled gold NPs onto the gaps between two microelectrodes. In principle, the capacitance or conductivity measurements can be used to determine the filled NPs and the bound target molecule, but the sensitivity is limited. To overcome it, they further put the device into a solution of Ag^+^ and hydroquinone, which caused the deposition of silver NPs to bridge the microelectrodes and lead to readily measurable conductivity changes. With this method, target DNA at concentrations as low as 500 fM can be detected with a point mutation selectivity factor of 10^5^:1.

Previous studies indicate that DNA molecules [176] and DNA-based supra-structures [177,178,179] conduct electricity, and therefore it is possible to utilize the fabricated DNA-based nanostructures for electrical biosensing applications. For example, Bell *et al.* for the first time synthesized DNA origami nanopores with a typical diameter of 13–18 nm [180]. After the created solid-state nanopore was assembled into a microfluidic measurement cell the current–voltage characteristic was tested in a buffer solution. The fabricated biosensor can be employed for the detection of λ-DNA molecules. This approach paves the way for the fabrication and sensing applications of the single-molecule nanopore sensors based on DNA nanostructures. At the same time, another research group also reported the synthesis of DNA origami gatekeepers and the application as solid-state nanopores [181]. The fabricated electrical nanopore sensor can be utilized for the label-free sensing of DNA molecules.

**Figure 8:**
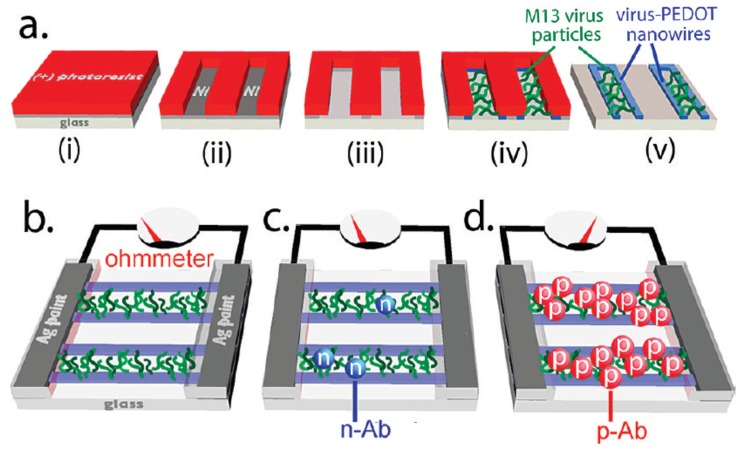
Virus-templated nanowires based electrical biosensor: (**a**) schematic preparation of virus-polymer nanowires; (**b**) detection mechanism of electrical biosensors; and (**c**,**d**) detection of antibodies (Reprinted with permission from [182], published by American Chemical Society, 2010).

Linear virus can be served as the excellent 1D template to connect the electrodes for the fabrication of electrical sensors. For example, Arter *et al.* reported the M13 bacteriophage based polymer nanowires for the electrical biosensing of antibodies [182]. Figure 8a shows the fabrication process of the linear arrays of M13-polymer hybrid nanowires. Firstly, they created a nickel film on glass substrate by the vapor deposition technique, and then coated the nickel film with a photoresist (step i). Secondly, they used a photolithography technique to create the desired position for the nanowires (step ii). Then, the oxidative degradation was utilized to remove the exposed nickel (step iii). After that, the etched substrate was used as the working electrode in a three-electrode cell for the electrodeposition of polymer nanowires and M13 virus NPs (step iv and v). The obtained M13-polymer nanowire device was further used for the resistance-based measurements to sensing antibodies, as shown in Figure 8b–d. It was found that the resistance revealed no change by adding the negative control antibody (Figure 8c), but the resistance showed significant change due to the presence of virus binding positive antibody (Figure 8d). The fabricated biosensor has obvious advantages like real-time, reagent-free, quick sensing, and environmentally friendly. In addition, the M13 bacteriophage is readily amenable to tailoring of its surface for molecular recognition using phage display, and therefore it is possible to find broad applications in clinical diagnostics with the introduced biosensor. Recently, Moon and co-workers further demonstrated that M13 bacteriophage can be used to organize gold and palladium NPs into linear arrays, which can be used as seeds for creating metallic nanowires by electroless deposition [183,184]. The fabricated electrical sensors show potential applications for sensing H_2_ and H_2_S.

Another typical electrical sensor is the field effect transistor (FET). Keren *et al.* for the first time reported the preparation of DNA-templated CNT-FET device [185]. The CNT-FET was assembled via a three-strand homologous recombination reaction between a long dsDNA (acts as a scaffold) and a short, auxiliary ssDNA. The assembly process was guided by the information encoded in these DNA molecules. They found that the DNA scaffold molecules provide the address for the precise localization of the CNTs and the template for the formation of extended metallic nanowires. Maune and co-workers reported the self-assembly of CNTs into 2D geometries by using DNA origami templates [186]. They found that the created CNT-DNA origami structures can be used to fabricate FET device, which shows stable FET-like behavior. In another case, Atanasova and co-workers reported the TMV-templated synthesis of ZnO nanowires for the fabrication of FET sensor [121]. Their study indicated that TMV does not only function as a structural scaffold for the nucleation and growth of ZnO nanowires, but also has an obvious effect on the properties of the formed semiconductor layer, such as the electron injection capability.

### 3.7. QCM Sensing

QCM has being one of the most promising label-free sensing techniques with high sensitivity and fast assay [187]. QCM-based sensors have been widely used for the detection of DNA, protein, virus, and other molecules [188]. Here, we would like to provide the recent key publications on the fabrication of QCM sensors by assembling biomolecular nanostructures on the QCM sensor chip [189,190,191,192,193].

**Figure 9:**
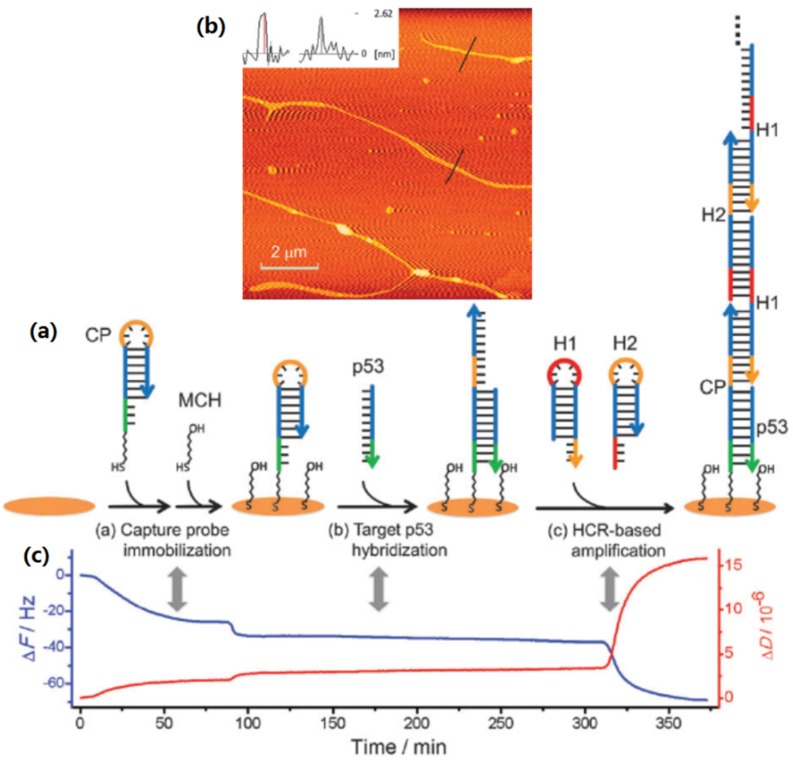
Self-assembled DNA nanostructure-based QCM biosensing of nucleic acids (**a**) Schematic presentation for the formation of DNA nanostructure for QCM biosensing; (**b**) Typical AFM image of the self-assembled DNA nanowires; (**c**) Corresponding QCM response curves related to the steps in (**a**) (Reprinted with permission from [189], published by Royal Society of Chemistry, 2012).

DNA-based nanostructures are the potential and efficient signal amplifiers for the QCM biosensing platform. Previously, Tang *et al.* demonstrated a self-assembled DNA nanostructure-amplified QCM with dissipation biosensing platform for the detection of nucleic acids [189], as shown in Figure 9. Figure 9a presents the fabrication and sensing mechanism of the self-assembled DNA nanostructure-based QCM sensor. Firstly, the capture probe (CP) and mercaptohexanol molecule (MCH) were immobilized on the gold chip (step a). The sequence of CP was designed precisely so that the initiator domain was protected by hairpin initially. Secondly, the target p53 was injected into the system for DNA hybridization (step b), which unfolded the closed CP hairpin structure to free the initiator domain. In this process, the low mass of target p53 is not enough for creating the clear frequency change, and therefore a signal amplification strategy is necessary. After that, two addition hairpins, H1 and H2, were designed and added into the chip to facilitate cross-hybridization on the free initiator domain (step c). Finally, the exposed initiator domain of CP triggered the hybridization chain reaction by the two hairpins into a 1D helix DNA nanostructure. The polymerized 1D helix DNA structure was measured with atomic force microscopy (AFM), and the typical height image is shown in Figure 9b. The corresponding QCM response curves (Figure 9c) in every steps of Figure 9a indicate that the formation of self-assembled DNA 1D nanostructure can greatly enhance the detection signals of p53 target. A detection limit of 0.1 nm for p53 was obtained. In another similar study, Altintas *et al.* developed a DNA-based QCM biosensor system for the detection of one-point mutation in TP53 gene [190]. A highly specific and sensitive hybridization reaction was successfully achieved in the concentration range of 0.03–2 µM detection probe with a rapid and real-time measurement.

DNA-NP hybrid nanostructures can also be utilized for the fabrication of QCM biosensing platforms. For example, previously Hao *et al.* reported a DNA probe and gold NP based QCM biosensor for the amplified detection of *Bacillus anthracis* [191]. Firstly, they immobilized a thiol DNA probe onto the gold chip of QCM, and then injected the longer target DNA molecule into the system to hybridize with the probe DNA. Finally, the DNA-functionalized gold NPs were added to hybridize with another part of the target DNA. The introduction of gold NPs into this QCM sensing platform can greatly amplify the frequency change, and a detection limit of 3.5 × 10^2^ CFU/mL was achieved for the detection of *Bacillus anthracis*. Recently, Li and co-workers fabricated a smart DNA-gold NP probe for detecting sing-base mutation in a p53 gene on the QCM platform by using the similar sensing mechanism [192]. A detection limit of about 35 pM was obtained.

DNA-protein hybrid nanostructure can also be the candidate to amplify the QCM signals for the low-concentrated sensing. Recently, Zhao *et al.* reported a facile strategy to *in situ* assemble DNA-streptavidin dendrimer nanostructure onto the gold chip and further fabricated a new amplified QCM platform for nucleic acid sensing [193]. The created sensing platform has the advantages like good designability and efficient mass amplification with label-free and real-time measurements. The detection limit is about 23 pM for sensing the p53 gene fragment with remarkable improvement in sensitivity. This amplification strategy based on the formation of DNA-streptavidin dendrimer nanostructure for QCM sensing has great potential to detect disease-related nucleic acids in biological samples.

## 4. Conclusions and Outlooks

The above presented examples indicate that biomolecules or microorganisms and their superstructures play very important roles on the synthesis of functional nanostructures and nanomaterials, which show the potential applications for the fabrication of colorimetric, florescent, SPR, SERS, EC, electrical, and QCM sensors. Firstly, biomolecules/microorganisms and their superstructure can provide 0, 1, 2, and 3D templates for the synthesis of desired nanomaterials. The preparation process is simple and controllable. Secondly, biomolecules/microorganisms have unique biological and biophysical properties, which are responsible for the biomimetic synthesis of hybrid nanomaterials. The biomimetic synthesis is facile and green. Thirdly, the introduction of biomolecules/microorganisms into the synthesized nanomaterials can improve the biological molecular recognition of the final materials. Therefore, the selectivity and sensitivity of the fabricated sensors can be greatly improved.

Here we still want to highlight several shortcoming of the biomimetic synthesis of hybrid nanostructures and nanomaterials. Firstly, it is hard to remove the biomolecular building blocks from the created nanostructures for electrical applications. For example, the biomolecular building blocks would have negative effects on the performance of conductive nanowire and nanodevices. Secondly, the biomimetic synthesis is not suitable for the large-scale synthesis of functional nanomaterials. For the practical applications in the future, the methods for the controlled synthesis of uniform bionanostructures and the optimal metallization or mineralization should be improved. Thirdly, the biomimetic synthesis of hybrid nanostructures is much expensive compared to the normal chemical synthesis. With the developments of biomolecules synthesis and extraction, this shortcoming could be overcome.

For the future developments of the synthesis and application of biomolecule-based nanostructures and nanomaterials, there are still some points that should be pointed out. Firstly, more attentions should be paid onto the design and fabrication of 3D DNA nanostructures, and the further modification of functional NPs and quantum dots for biomedical applications. Secondly, functional peptide nanofibers with designed functional motifs should be used for the synthesis of 1D, 2D, and 3D nanomaterials by self-assembly. In addition, the combination of peptide nanostructures with NPs may create more functional bionanomaterials for cell culture and drug delivery. Thirdly, other attentions should also be focused on the combination of biomolecular nanostructures with carbon materials like CNT and graphene, which will improve the applications in EC sensing, EC catalysis, and energy storage.
